# Effects of repeated low-level red light on refractive development during childhood: a systematic review and dose–response meta-analysis up to 12 months

**DOI:** 10.3389/fmed.2025.1657295

**Published:** 2025-12-10

**Authors:** Qi Ren, Zhe Chu, Zhengzhi Qiu, Hao Cheng, Lin Liu

**Affiliations:** 1 Department of Ophthalmology, The First Affiliated Hospital, Guangzhou Medical University, Guangzhou, China; 2 Department of Ophthalmology, The First Affiliated Hospital, Sun Yat-sen University, Guangzhou, China; 3 Shandong Provincial Key Laboratory of Ophthalmology, Eye Institute of Shandong First Medical University, Qingdao, China

**Keywords:** repeated low-level red light, refractive development, myopia, corneal curvature radius, axial length, spherical equivalent refractive error

## Abstract

**Objective:**

This study aimed to systematically evaluate the effects of longitudinal repeated low-level red light (RLRL) intervention on refractive development in children over a period of up to 12 months.

**Methods:**

A systematic search was conducted across four electronic databases, including randomized controlled trials involving RLRL interventions for children. The data covered literature from the establishment of each database up to August 2024. Dose–response meta-analyses were performed using a random-effects model to evaluate the impact of RLRL on refractive development at 1, 3, 6, and 12 months.

**Design:**

The research is a systematic review and meta-analysis of randomized controlled trials.

**Data sources:**

Data were searched across PubMed, Embase (Ovid), Web of Science (WoS), and the Cochrane Central Register of Controlled Trials (CENTRAL) databases from their inception to 3 August 2024.

**Eligibility criteria for selecting studies:**

Randomized controlled trials comparing RLRL with other pharmacological interventions or no treatment for refractive development in children were included. Independent data extraction was conducted, and study quality was assessed. A meta-analysis was carried out using random-effects models to evaluate the effects of RLRL on refractive development outcomes compared with control groups.

**Main outcomes:**

The main outcomes included anterior corneal curvature, axial length (AL), and spherical equivalent refractive error (SER).

**Results:**

In total, 12 unique RCTs were included. For corneal curvature, evidence for interventions with RLRL treatment being flatter than comparators was lacking (SMD = −0.01, 95%*CI* −0.19 to −0.12; *I*^2^ = 0, *p* = 0.76). RLRL treatment was superior to comparators for both AL (SMD = 0.27, 0.06, 0.14, 0.19, 95%*CI* 0.20–0.33, 0.05–0.08, 0.11–0.17, 0.16–0.23; *I*^2^ = 91, 75, 85, 85%, *p* < 0.01, respectively) and SER (SMD = 0.62, 0.03, 0.18, 0.32, 95%*CI* 0.46–0.77, −0.03 to 0.08, 0.12–0.24, 0.24–0.40; *I*^2^ = 91, 75, 85, 85%, *p* = 0.05 for the 6th month and <0.01 for the remainder) in slowing the progression of myopia during 1, 3, 6, and 12 months. According to the dose–response meta-analysis, the effects of RLRL on AL and SER began to decline after 3 months of treatment.

**Conclusion:**

RLRL treatment exhibited a non-linear trend, suppressing axial length development and mitigating spherical equivalent refraction severity, with intervention efficacy diminishing by the third month, and the evidence regarding its effectiveness in flattening anterior corneal curvature remains inconclusive.

**Systematic review registration:**

https://www.crd.york.ac.uk/PROSPERO/, Identifier: CRD42024575823.

## 1 Introduction

Visible red light, typically with an energy density value of 0.5–4.0 J/cm^2^ and a visible spectrum ranging from 600 to 700 nm, is helpful in enhancing the proliferation rate of various cell lines (1). It has been widely used in clinical treatment of diseases in many fields, including dermatology (2) and pain management (3). Evidence from randomized controlled trials (RCTs) has shown the efficacy and safety of repeated low-intensity red light (RLRL, typically with a wavelength of 650 nm) therapy for controlling myopia in children and adolescents (4). Therefore, RLRL treatment has been considered as a strategy to prevent the occurrence and delay the progression of myopia. For example, a multicenter RCT of RLRL irradiation for myopia control in school-age children was conducted in China, providing 1-year high-level evidence of the technique’s efficacy, safety, and subject compliance in adjuvant myopia treatment (5), and the corresponding expert consensus was published (6).

Existing systematic reviews and meta-analyses of RLRL (4, 7–12), which primarily concentrate on alterations in AL and choroidal blood flow, underscore the critical importance of inhibiting AL elongation as a pivotal research avenue for managing axial myopia and its associated complications. These high-quality reviews furnish robust evidence substantiating the efficacy of RLRL in mitigating both the onset and progression of axial myopia, thereby reinforcing the research paradigm positing that RLRL attenuates scleral hypoxia-induced extracellular matrix remodeling. Furthermore, several previous systematic reviews have evaluated the effectiveness and safety of the RLRL therapy in slowing the progression of myopia in children and adolescents (4, 8, 10–13) and reported that RLRL therapy can inhibit the growth of AL and slow down the progression of SER change.

In addition to SER and AL for assessing myopia severity, corneal refractive power, which contributes approximately two-thirds of the eye’s total optical power, represents a critical component for understanding RLRL’s mechanism. Recent research has revealed that intrinsically photosensitive retinal ganglion cells (ipRGCs) serve as a regulatory hub orchestrating coordinated ocular growth during emmetropization (14). These cells integrate two distinct light signals with opposing effects on refractive development: the melanopsin-mediated intrinsic signal (maximally sensitive to blue light, approximately 480 nm) drives axial elongation toward myopic shift, whereas the rod/cone-mediated extrinsic signal promotes corneal flattening (increased curvature radius) toward hyperopic shift. Intriguingly, the clinical effects of RLRL align closely with predictions from this dual-pathway model—red light selectively activates the rod/cone pathway while minimally stimulating melanopsin, potentially inducing corneal flattening to counteract myopic progression (14, 15). This mechanistic framework predicts coordinated changes in both corneal curvature and axial length during RLRL treatment. However, while existing clinical studies have consistently demonstrated RLRL’s efficacy in controlling axial elongation, systematic evaluation of the model-predicted corneal curvature changes remains limited. To validate this mechanistic hypothesis, examining corneal curvature alterations in clinical trials is essential to confirm the ipRGC-mediated dual-pathway model.

Therefore, this systematic review and dose–response meta-analysis aimed to comprehensively evaluate whether RLRL treatment induces the theoretically predicted coordinated changes in corneal curvature, AL, and SER based on available RCT evidence over a 12-month period.

## 2 Methods

### 2.1 Protocol and guidance

This systematic review and meta-analysis was performed in accordance with the Preferred Reporting Items for Systematic Reviews and Meta-Analyses (PRISMA) guidelines (16). The protocol for this review was developed and prospectively registered with PROSPERO (CRD42024575823).

### 2.2 Eligible criteria

Eligible studies were randomized controlled trials (RCTs) that met the following criteria: (1) Participants were children younger than 16 years old. Given the absence of current evidence indicating a node between processes of emmetropization and myopia development, we included patients diagnosed with or without myopia, without any ocular comorbidities including strabismus and amblyopia, or surgical interventions for myopia correction, e.g., refractive surgery. (2) The intervention group received RLRL treatment to control myopia progression. The comparator group received a placebo or any other intervention that did not involve light therapy (e.g., pharmacological treatments or single-vision spectacles). We imposed no restrictions on the treatment duration or dosage.

Studies in groups receiving orthokeratology were excluded, as cornea compression could affect the eye’s total refractive power. Based on available research (17), wearing single-vision spectacles (SVSs) is not considered a parallel intervention; moreover, SVSs help ensure that the red light enters the eyes properly.

### 2.3 Outcomes

Eligible studies needed to extract at least one of the outcomes of interest as follows:

#### 2.3.1 Anterior corneal curvature

Anterior corneal curvature refraction (CR), which is frequently represented as mean keratometry (Km) in ophthalmic studies, is equivalent to the average of flat (K1) and steep (K2) keratometry for the anterior cornea. CR was also extracted as one form of corneal curvature. When post-intervention Km values were available in the study with baseline change Km values, we preferred to extract post-intervention values.

#### 2.3.2 AL and SER

We reviewed the baseline with mean changes (ΔAL and ΔSER). If ΔAL and ΔSER were not included in the study, we would prioritize the AL and SER.

### 2.4 Search strategy

One of the authors (QR) conducted the search of several databases: PubMed, Embase (Ovid), Web of Science (WoS), and the Cochrane Central Register of Controlled Trials (CENTRAL) from inception to 3 August 2024. We also searched ClinicalTrials.gov and the World Health Organization International Clinical Trials Registry Platform to identify ongoing or unpublished eligible trials. To maximize the search for relevant articles, we reviewed reference lists of identified trials and systematic reviews. There were no language limitations. Only human studies available with full text were analyzed. Supplementary file 1 presents the search strategy.

### 2.5 Study selection and data collection process

After duplicate records were removed, two independent researchers (QR and ZC) screened all titles and abstracts. Full texts were obtained and further assessed when studies appeared eligible. Disagreements were resolved by consensus.

The same researchers (QR and ZC) used a prespecified, piloted data extraction form to collect information from the included trials in accordance with the protocol of this study, including population and intervention characteristics and outcome data (data from baseline to 12 months). The data were extracted by one reviewer (QR) and verified by another reviewer (ZC). Differences were resolved in consultation with the third reviewer (ZZQ). When RCTs had more than two arms, we pooled data from the separate treatment arms. We extracted the variables from the table with priority and then from the graphical figure using GetData Graph Digitizer (version 2.25.0.32). For studies that were eligible for inclusion but did not provide enough data for meta-analysis, we synthesized the study results narratively. Disagreements were resolved by consensus; if they could not be resolved, the study would be excluded.

### 2.6 Assessment of risk of bias and quality of evidence

Two researchers (ZZQ and LL) independently assessed the quality of all included trials using the Cochrane Collaboration risk of bias tool (18). They also examined the quality of evidence for outcomes using the Grading of Recommendations Assessment, Development, and Evaluation (GRADE) approach. Disagreements were discussed between the reviewers until a consensus was reached.

### 2.7 Data synthesis and analysis

For data extraction, the priority was based on article description, followed by graphic information, and finally by the measurement in the figure through the GetData Graph Digitizer software. We extracted the mean and standard deviation (SD) for outcome measures. If the baseline or outcome data were expressed in a study as a median and quartile range (IQR) or as a mean and 95% confidence interval (95% CI), we converted these data to mean ± standard deviation (19).

Km is a key measurement of anterior corneal curvature refraction. However, some studies reported K1 and K2, for which we conducted subgroup analyses, respectively.

For AL and SER, the ‘mean ± SD’ values of ΔAL and ΔSER were calculated by the baseline values with the mean changes, following the principles outlined in the Cochrane Handbook for Systematic Reviews of Interventions (20). The calculation was performed as follows:


$$\mathrm{mea}n_{\mathrm{AL}}-\mathrm{mea}n_{AL_{\text{baseline}}}\pm \sqrt{\begin{array}{l} s{d^{2}}_{\mathrm{AL}}+s{d^{2}}_{AL_{\text{baseline}}}-\\ \left(2\times r\times sd_{\mathrm{AL}}\times sd_{AL_{\text{baseline}}}\right) \end{array}}$$

$$\mathrm{mea}n_{\mathrm{SER}}-\mathrm{mea}n_{\mathrm{SE}R_{\text{baseline}}}\pm \sqrt{\begin{array}{l} s{d^{2}}_{\mathrm{SER}}+s{d^{2}}_{\mathrm{SE}R_{\text{baseline}}}-\\ \left(2\times r\times sd_{\mathrm{SER}}\times sd_{\mathrm{SE}R_{\text{baseline}}}\right) \end{array}}$$

Mean and sd represent the mean and standard deviation of each variable, and *r* represents the correlation between the values. If the extraction of *r* is not available in the research, it will be treated as zero in this review.

Four follow-up groups (1 month, 3 months, 6 months, and 12 months) were included in this meta-analysis. If a study reported outcomes at multiple follow-up time points, each outcome would be separately incorporated in the corresponding follow-up group.

The two reviewers (QR and ZC) independently rated the certainty of evidence for each outcome using the Grading of Recommendations Assessment, Development, and Evaluation (GRADE). The certainty of the evidence was assessed for the selection, performance, detection, attrition, reporting, and other biases if they existed. We used the Cochrane *Q* test to identify heterogeneity and quantified it using the *I*^2^ statistic and the between-study variance *τ*^2^. We followed Cochrane Handbook recommendations to interpret *I*^2^ values (<0.4 representing a small effect, 0.4–0.7 a moderate effect, >0.7 a large effect) (20). Funnel plots and Egger’s test were conducted to identify small studies and publication bias. The fixed-effect model was used for outcomes of K1 and K2, and the random-effect model was used for outcomes of AL and SER. Dose–response relationships of AL and SER variations across months were analyzed using a restricted cubic spline (RCS) regression model. Nodes were set at the 5th, 35th, 65th, and 95th percentiles of the dose distribution, and turning points were identified through numerical differentiation by calculating the second-order derivatives. For all statistical analyses, we considered an *α* < 0.05 to be statistically significant. Review Manager (version 5.4), R (version 4.5.0), and STATA (version MP 18.0) were used for all analyses.

## 3 Results

**Figure 1:**
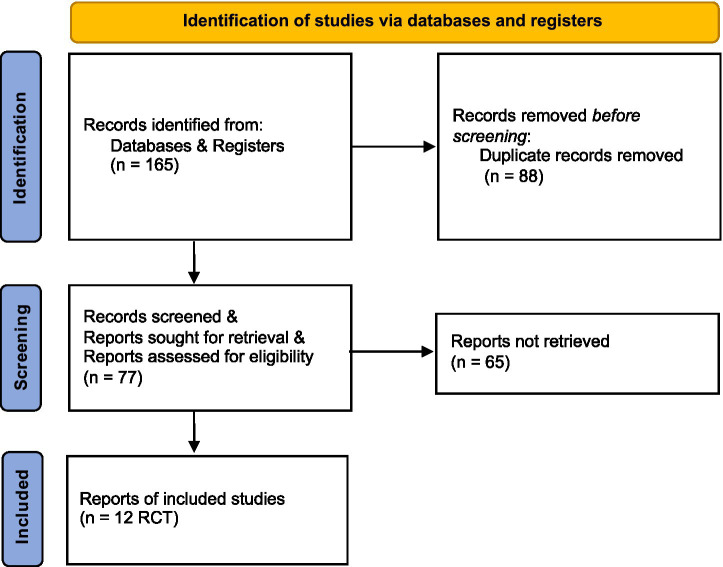
PRISMA flowchart of paper selection. PRISMA flowchart outlining the search strategy and details on the studies finally included in the meta-analysis.

A literature search on 3 August 2024 identified a total of 165 unique records, of which 14 full texts were assessed for eligibility. The main reasons for exclusion at the full-text stage were that studies did not meet the eligibility criteria of this review. Two studies could not be included in this meta-analysis because information to permit calculation of means or SDs was insufficient (21, 22). In total, 12 unique RCTs were included (5, 23–33). Due to the limited quantity of articles, a prospective non-RCT was incorporated into the study on anterior corneal curvature (34). PRISMA flow diagrams show the search results (Figure 1) (35). Regarding safety, all 12 included RCTs explicitly reported adverse event monitoring. Across all studies, no treatment-related adverse events were reported, and zero participants withdrew due to safety concerns.

### 3.1 Characteristics of the included studies

During the sample size extraction process, article descriptions, flow diagrams, and baseline tables were checked simultaneously, and if there were conflicts, the results were finally confirmed by checking the number and percentage of gender. Supplementary Table 1 provides characteristics of the included studies. Study sample sizes varied from 59 to 336 participants (23, 27). There were no statistically significant differences in the baseline characteristics of the included children. The majority of the included studies were conducted in China (5, 23–26, 28–33); another study was conducted in Melbourne, Australia (27).

The mean light wavelength of the included studies was 650 nm, and the prescribed frequency was set at 3 min twice daily, with a minimum interval of 4 h between sessions; however, the duration varied slightly, ranging from 5 days per week to daily administration. In addition, the power output of the light sources varied; while all remained within the certified safe power range, in a study by Zhou et al. (36), the light source had three different power settings: power of 0.37 ± 0.02 mW, 0.60 ± 0.2 mW, and 1.20 mW, which effect sizes were combined as three separate groups in this review.

The majority of interventions of RLRL were compared to no intervention (*n* = 11, 91.6%), and the use of SVS was not regarded as a parallel intervention. One study (*n* = 1, 8.3%) included an active comparator using low-dose atropine (25), which also met the inclusion criteria.

### 3.2 Risk of bias and quality assessment

In the included studies, the majority were judged to have an overall low risk of bias. The main reason for a high risk of bias judgment was the lack of blinding of participants and personnel (performance bias), as both participants and researchers were aware of the allocation. Supplementary figures present details of the risk of bias assessments for individual studies. The funnel plot appeared symmetrical among studies included in the meta-analysis of corneal curvature, AL, and SER, suggesting a low likelihood of publication bias (Supplementary figures).

### 3.3 Effect of RLRL on corneal curvature

**Figure 2:**
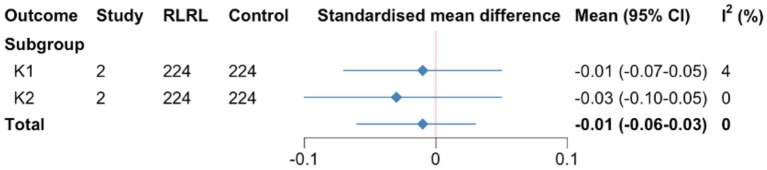
Summary SMD for corneal curvature meta-analysis. SMD, standardized mean differences; CR, curvature refraction. K1 and K2, flat and steep keratometry.

For K1 and K2, evidence for interventions with RLRL treatment being flatter than comparators was lacking (SMD = −0.01, 95%*CI* −0.19 to −0.12; *I*^2^ = 0, *p* = 0.76). (Supplementary figures; Figure 2).

### 3.4 Effect of RLRL on AL and SER

**Figure 3:**
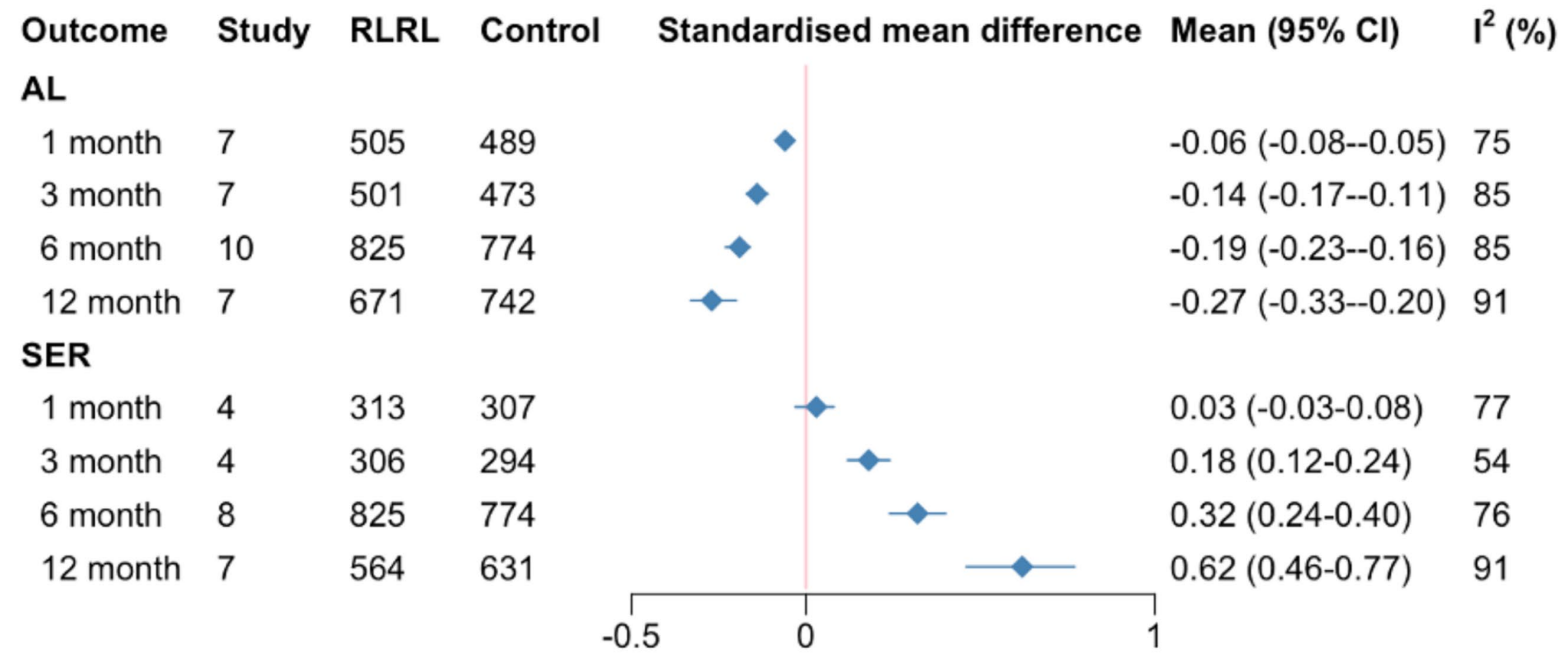
Summary SMDs for meta-analysis of AL and SER at each follow-up time. SMD, standardized mean differences; AL, axial length; SER, spherical equivalent refractive error.

In general, RLRL treatment was superior to comparators for both AL (SMD = 0.27, 0.06, 0.14, 0.19, 95%*CI* 0.20–0.33, 0.05–0.08, 0.11–0.17, 0.16–0.23; *I*^2^ = 91, 75, 85, 85%, *p* < 0.01, respectively) and SER (SMD = 0.62, 0.03, 0.18, 0.32, 95%*CI* 0.46 to 0.77, −0.03 to 0.08, 0.12–0.24, 0.24–0.40; *I*^2^ = 91, 75, 85, 85%, *p* = 0.05 for the 6-month and <0.01 for the remainder) in slowing the progression of myopia during 1, 3, 6, and 12 months (Supplementary figures). The influence of RLRL on the two subgroups is summarized in Supplementary figures. One study reporting AL and SER outcomes could not be included in the meta-analysis because it failed to report SDs. The study reported that the eyes of the children with RLRL treatment became less myopic based on the growth of AL and progression of SER (22) (Figure 3).

### 3.5 Predicted response in AL and SER of up to 12 months’ duration

**Figure 4:**
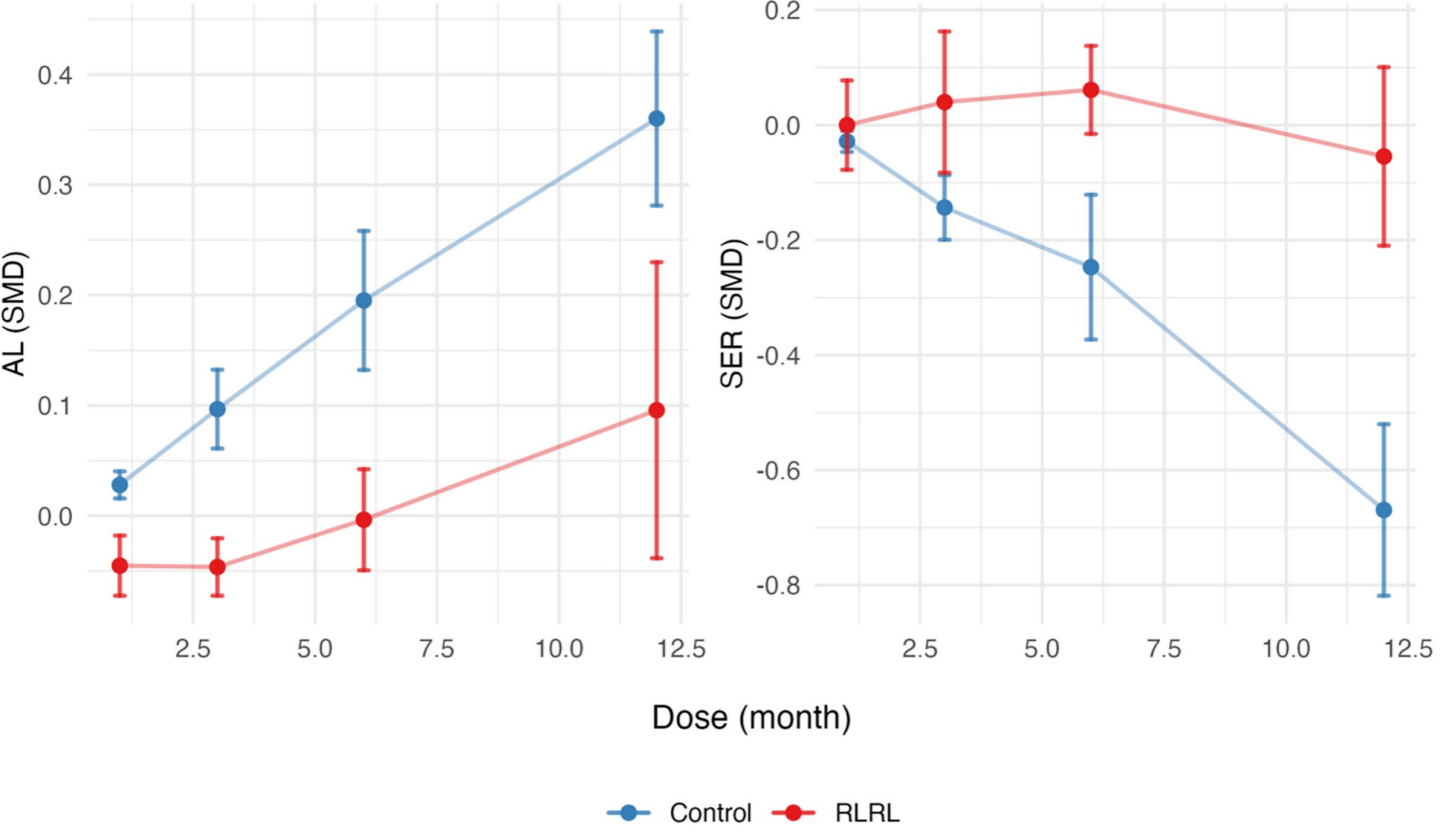
SMDs for AL and SER across months for the RLRL and control groups. Error bars represent the standard deviation. SMD, standardized mean differences; AL, axial length; SER, spherical equivalent refractive error.

**Figure 5:**
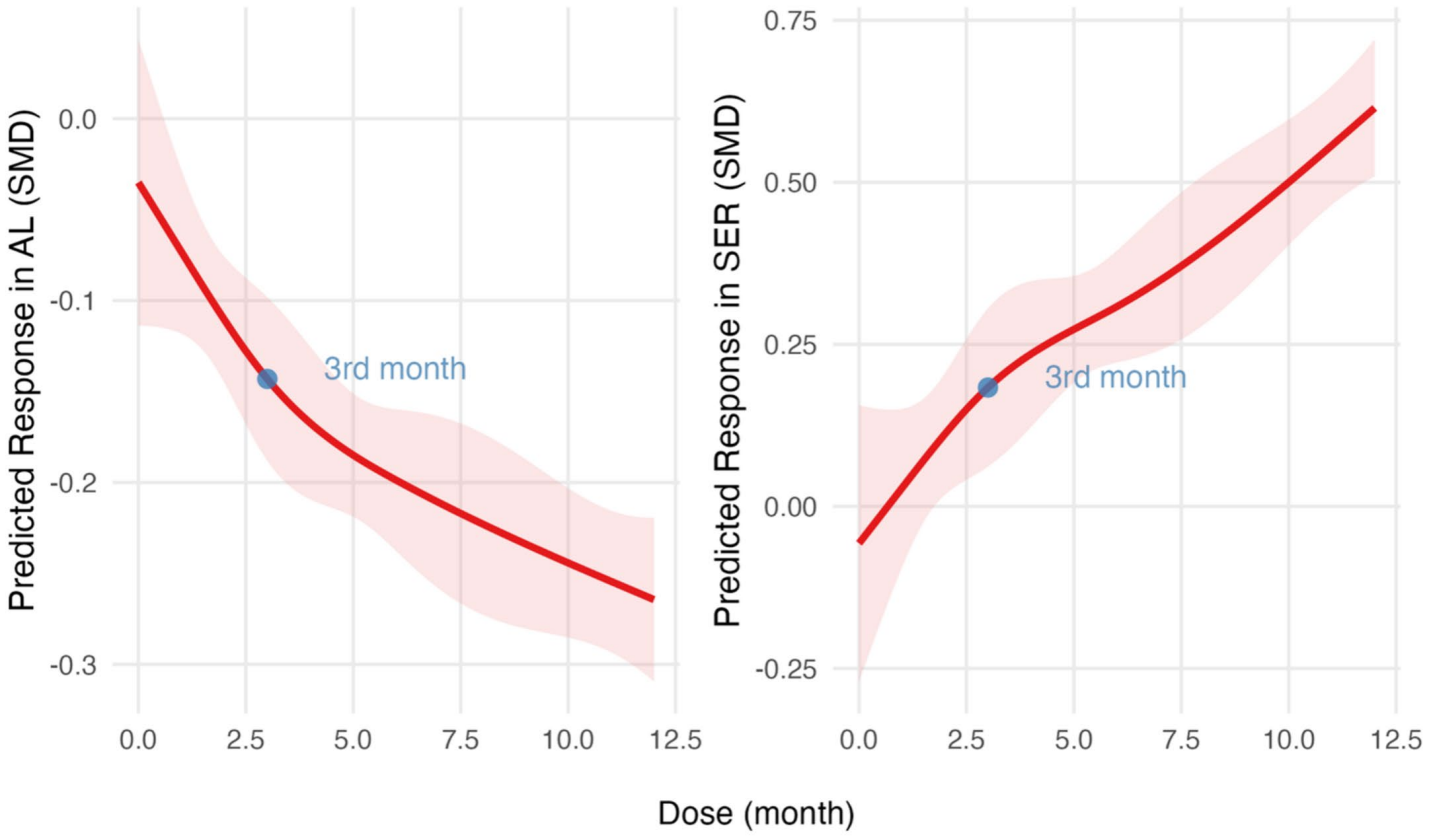
Dose–response relationship analyzed using restricted cubic spline regression models. SMD over time for AL (left) and SER (right). The red lines indicate predicted response trends, and the light red shaded areas represent 95% confidence intervals. The blue points mark the key inflection point. SMD, standardized mean differences; AL, axial length; SER, spherical equivalent refractive error.

The SMD slope is relatively gentle for AL and SER in the RLRL group, while the development trend of the control group remains stable (Figure 4). The RCS regression model fitting results indicate that the *R*^2^ for the axial length model is 0.54, with an *F*-statistic of 13.65 (*p* < 0.001). For the refractive power model, the *R*^2^ is 0.69, with an *F*-statistic of 20.62 (*p* < 0.001). These results support the significant non-linear effect of RLRL intervention on the refractive development during childhood (Supplementary file 2; Figure 5).

## 4 Discussion

Overall, this review included 12 RCTs examining the effects of RLRL on corneal curvature, AL, and SER in children compared to non-phototherapeutic interventions over a 1-year period. Two studies related to the primary outcomes of K1 and K2 (23, 32). The evidence showed that RLRL treatment suppressed axial development and reduced SER severity. However, no significant differences were found in anterior corneal curvature refraction between the RLRL and control groups after 1 year.

At present, the mechanism of preventing myopia occurrence and delaying myopia progression by RLRL is still unclear. The academic community generally acknowledges that visual information from near work and other close-range visual tasks can disrupt the retinal dopamine homeostasis, leading to a decrease in choroidal blood flow and subsequent hypoxia in the sclera. This hypoxic state is believed to induce remodeling of the scleral extracellular matrix, a process associated with myopia development. Children and adolescents undergoing RLRL exhibit increased choroidal thickness (36), suggesting that RLRL therapy stimulates retinal dopamine secretion, thereby enhancing choroidal blood flow. Thus, RLRL is believed to alleviate scleral hypoxia and prevent myopia-related scleral extracellular matrix remodeling (37, 38). However, this hypothesis encounters difficulties in explaining why monochromatic red light therapy demonstrates more pronounced effects in myopia control compared to extending outdoor light exposure (39, 40), particularly given that a significant portion of children experience marked reductions in axial length (AL) following treatment.

Recently, with the proposition of a dopamine-independent mechanism of myopia formation (14), new insights have emerged regarding the interpretation of the mechanisms underlying the retardation of myopia onset and progression by RLRL therapy. ipRGCs, constituting the third class of photoreceptors apart from rods and cones, are located in the inner layer of the retina. These cells function as output neurons, akin to other retinal ganglion cells, but uniquely contain the light-sensitive pigment melanopsin. Consequently, upon light exposure, the response of ipRGCs encompasses two components: one driven by signals from photoreceptors they receive and another generated by the activation of their intrinsic melanopsin. The “melanopsin-driven signals” and “rod/cone-driven signals” of ipRGCs independently influence refractive development by affecting AL and CR, respectively. When the retina is stimulated with red light, red cones are activated, while the activation of melanopsin (primarily sensitive to blue light) is minimal. In such cases, the photoreceptor-mediated signals on ipRGCs are enhanced, leading to an increase in CR. Therefore, in the context of RLRL therapy with red light stimulation, the red light-sensitive “rod/cone-driven signals” of ipRGCs are activated, while the blue light-sensitive “melanopsin-driven signals” are inhibited. RLRL therapy may regulate downstream signals transmitted by ipRGCs through these two upstream events, thereby inhibiting AL elongation and increasing corneal CR. Based on this hypothesis, RLRL not only promotes flatter corneal curvature but may also impact the compensatory adjustment of refractive power. Recent research findings suggest an interaction between myopiagenic hyperopic defocus and ipRGC signaling, indicating that proper ipRGC function may be crucial for normal refractive development and preventing the progression of myopia (41). Consequently, evidence regarding the effectiveness of RLRL treatment in flattening the cornea compared to control groups is currently insufficient due to of studies available for inclusion. Given that the “rod/cone-driven signal” of ipRGCs may lead to a reduction in corneal curvature under red light, further reports with long-term data validation are needed to fully understand the effect of RLRL on CR.

The limitations of this review primarily manifest in the insufficient evidence regarding the effects of RLRL intervention on CR development, due to the limited number of studies available for inclusion. Additionally, some of the incorporated studies were impacted by the COVID-19 pandemic. Although certain studies differentiated between interrupted and continuous intervention groups to minimize this effect, it cannot be definitively ruled out that the intervention effects were compromised to some extent. Finally, the optimal parameters for RLRL therapy remain undetermined. As more data become available, systematic aggregation and synthesis of RLRL parameter data are needed to identify protocols that balance efficacy and safety and to provide an evidence base for standardizing RLRL therapy.

In summary, while this review aimed to comprehensively synthesize the systematic effects of RLRL treatment on refractive development during childhood, the certainty of evidence regarding the effectiveness of RLRL in flattening anterior corneal curvature remains inconclusive. The available evidence supports that, compared to low-dose atropine or no intervention, RLRL treatment suppressed axial development and reduced SER severity. However, this effect exhibited a non-linear trend, with the intervention’s efficacy diminishing by the third month of accumulation.

## Data Availability

The original contributions presented in the study are included in the article/Supplementary material, further inquiries can be directed to the corresponding author.
