# Supplementary material for: Effects of repeated low-level red light on refractive development during childhood: a systematic review and dose–response meta-analysis up to 12 months
Source: Front Med (Lausanne). 2025 Dec 10;12:1657295. doi: 10.3389/fmed.2025.1657295 (PMC12728021; doi:10.3389/fmed.2025.1657295)
Supplement: Supplementary file 1 [file Data_Sheet_1.pdf]

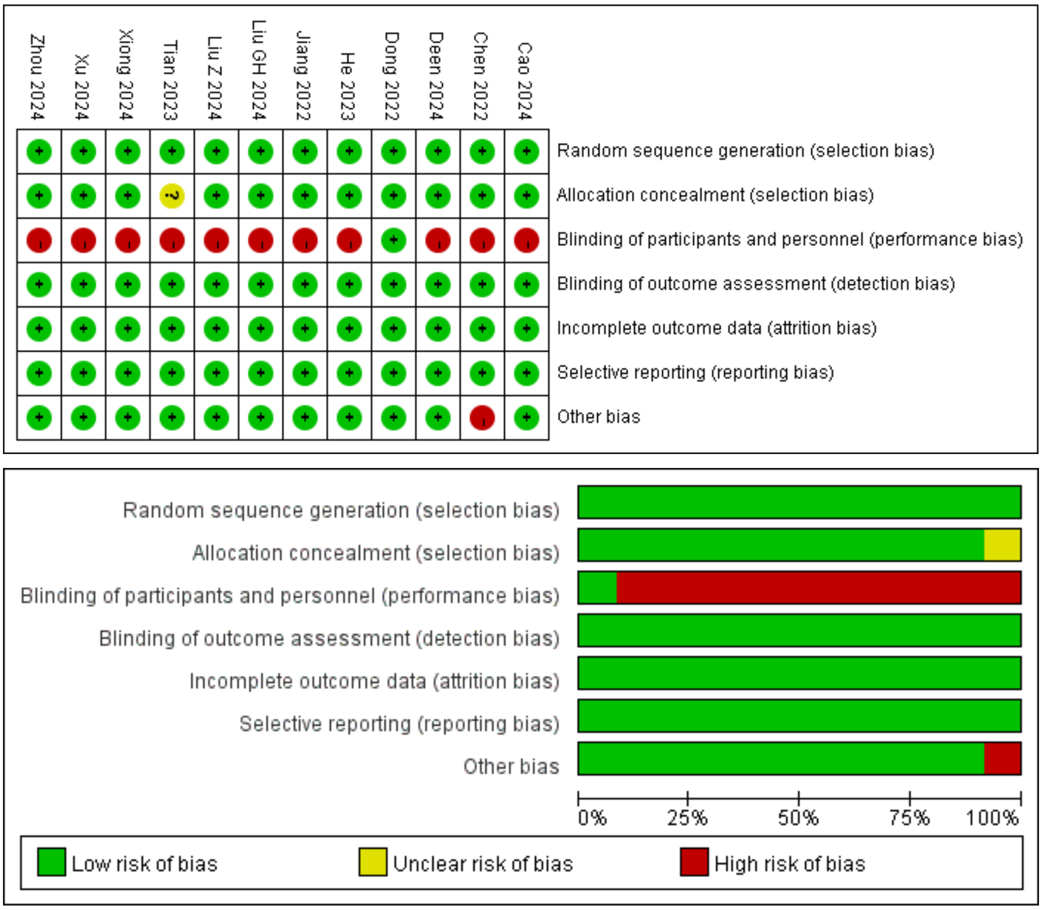

Supplementary figure 1. Summary of the risk of bias assessment. Demonstrates the risk of bias assessment for each trial through the utilization of the Cochrane risk of bias tool. The green-colored symbol indicates a low risk of bias, the yellow one represents an unclear risk of bias, and the red one corresponds to a high risk of bias.

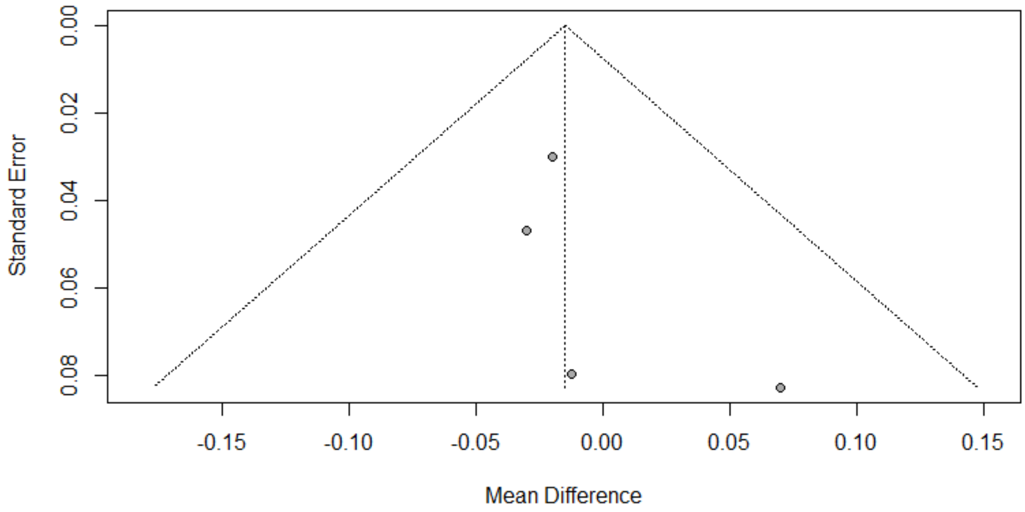

Supplementary figure 2. Funnel plots of K1 & K2.

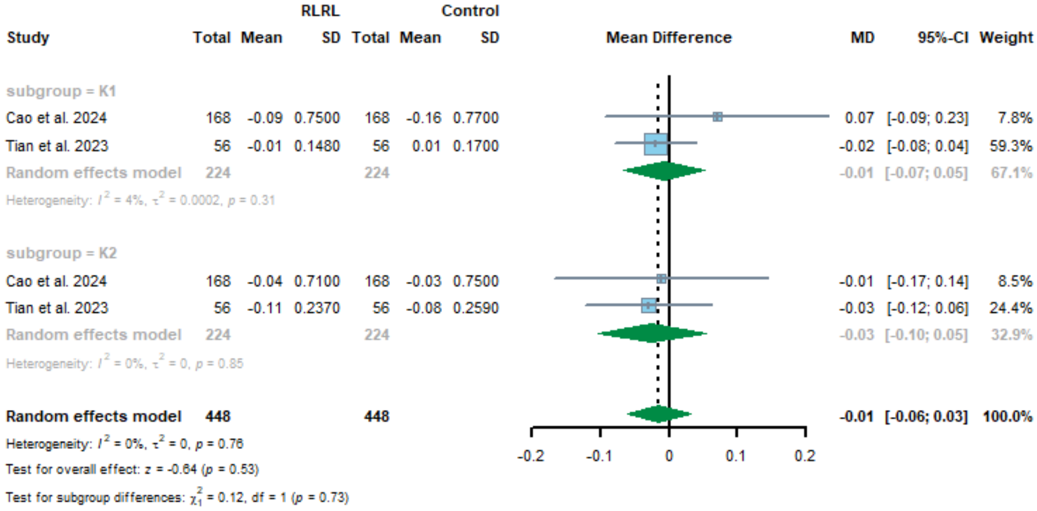

Supplementary figure 3. Forest plots of K1 & K2.

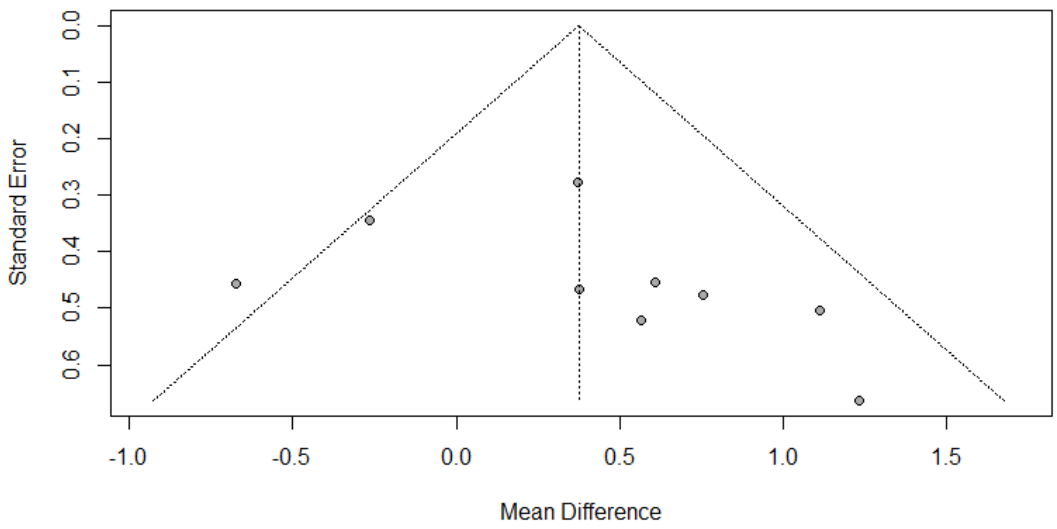

Supplementary figure 4a. Funnel plots of AL for 1 month.

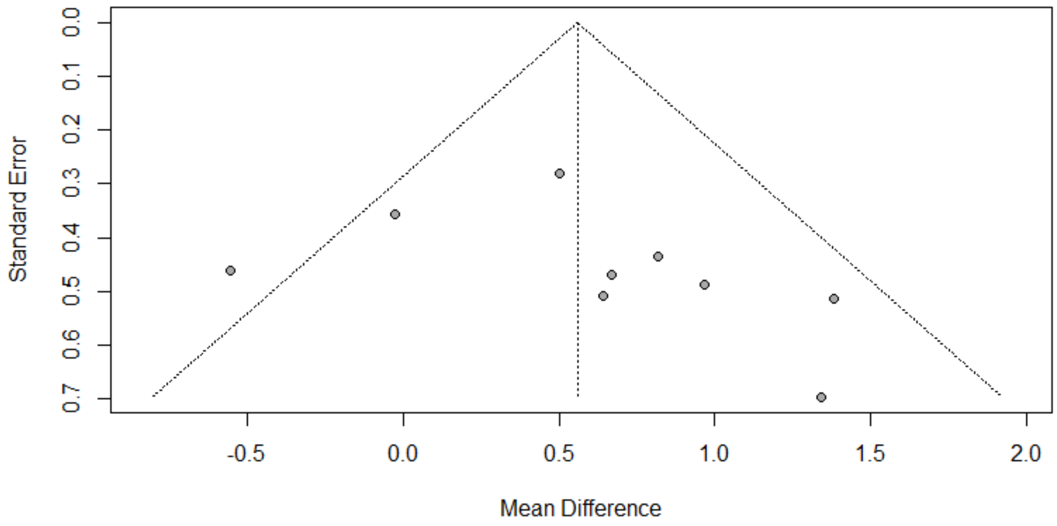

Supplementary figure 4b. Funnel plots of AL for 3 month.

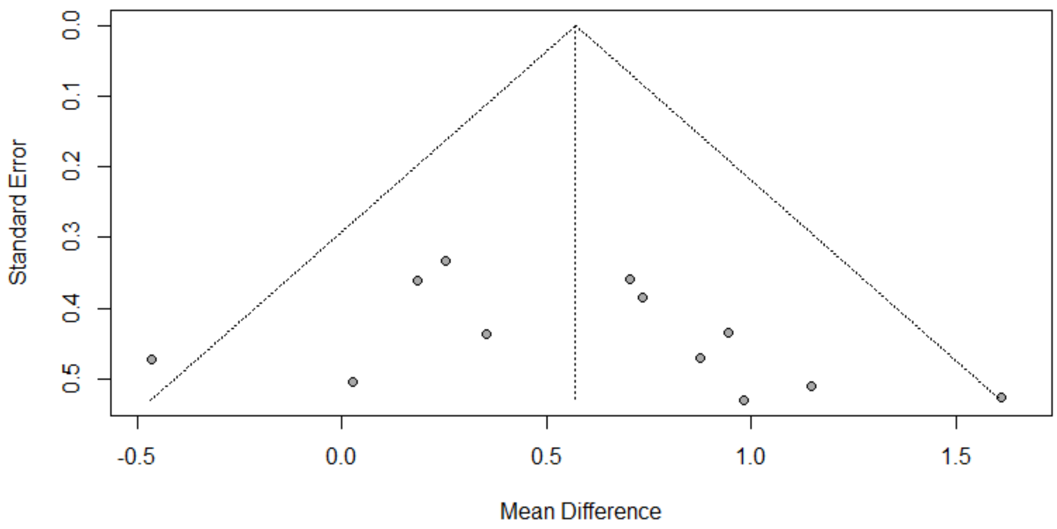

Supplementary figure 4c. Funnel plots of AL for 6 month.

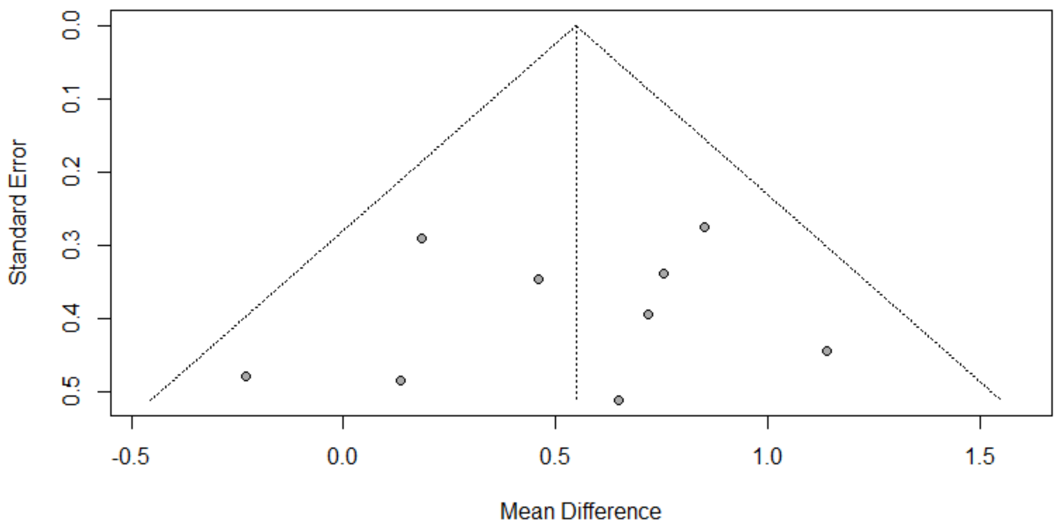

Supplementary figure 4d. Funnel plots of AL for 12 month.

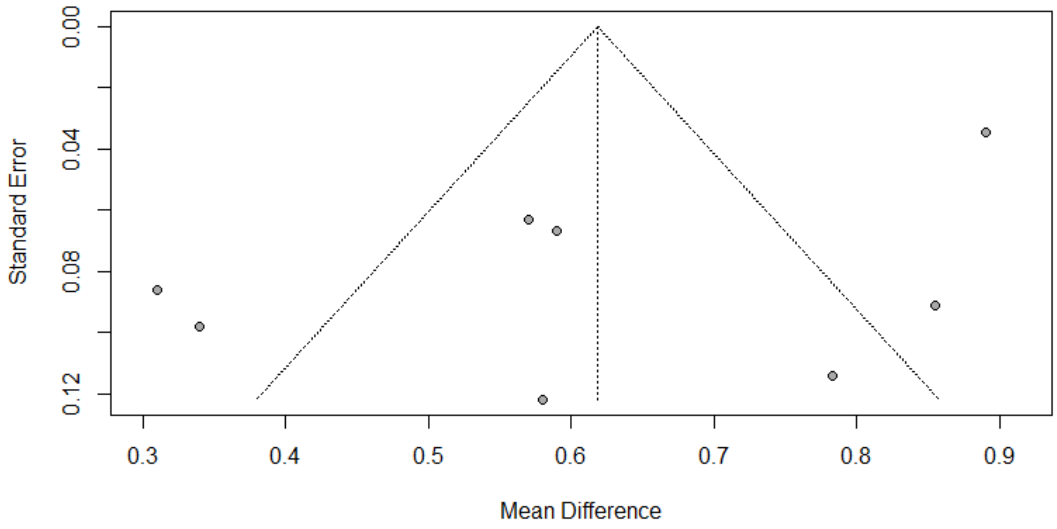

Supplementary figure 4e. Funnel plots of AL for 1 month.

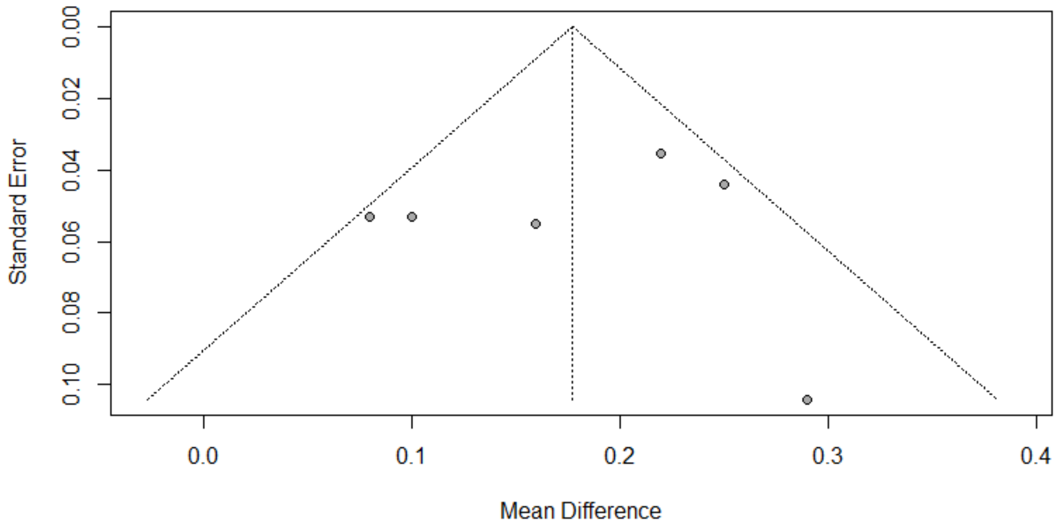

Supplementary figure 4f. Funnel plots of AL for 3 month.

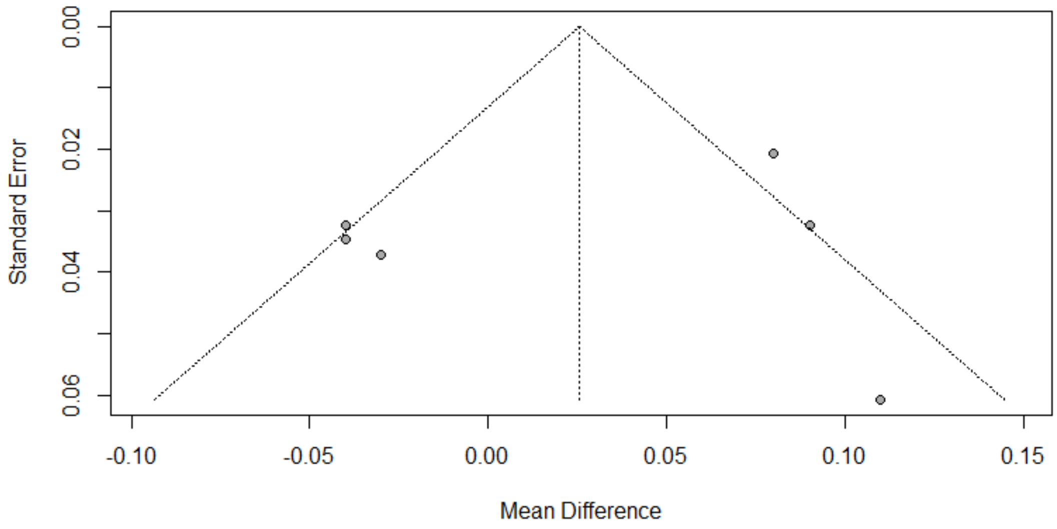

Supplementary figure 4g. Funnel plots of AL for 6 month.

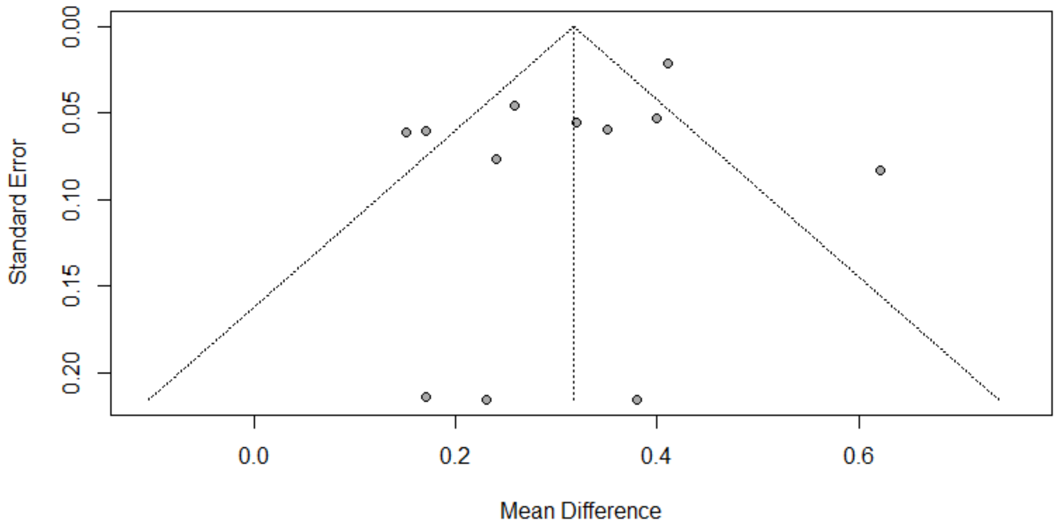

Supplementary figure 4h. Funnel plots of AL for 12 month.

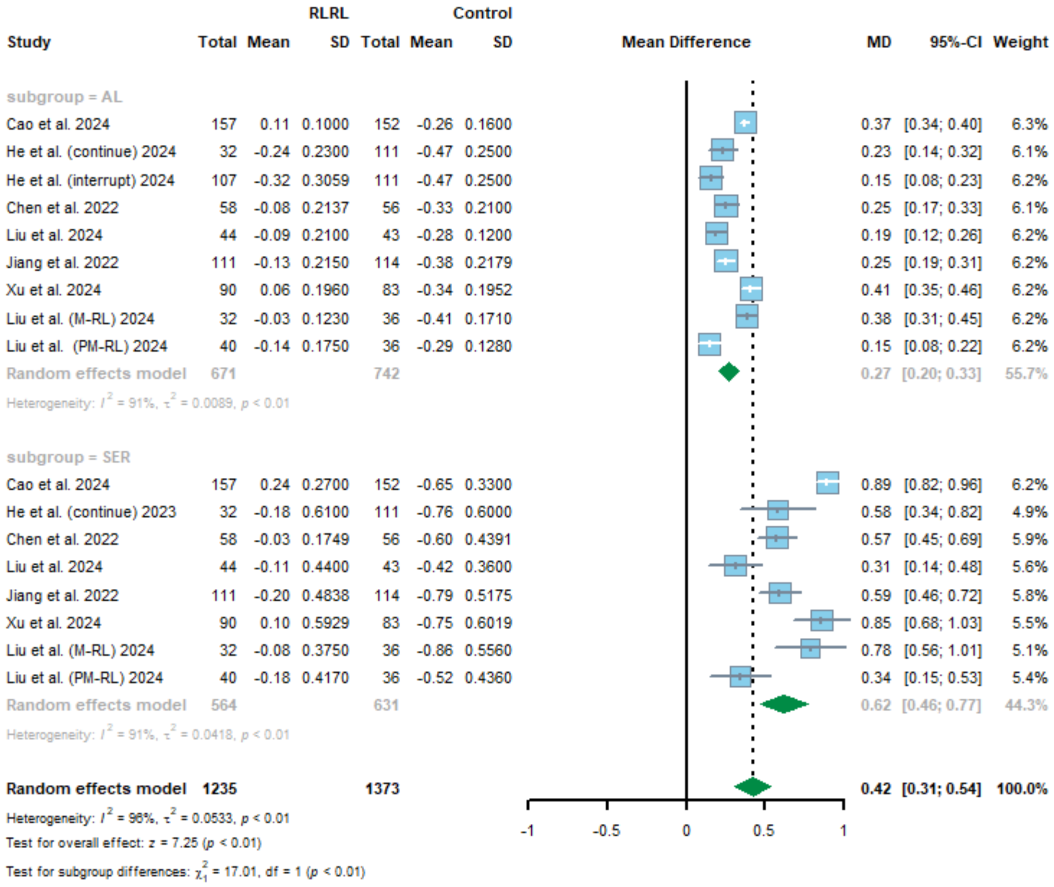

Supplementary figure 5a. Forest plots of AL and SER for 1 month.

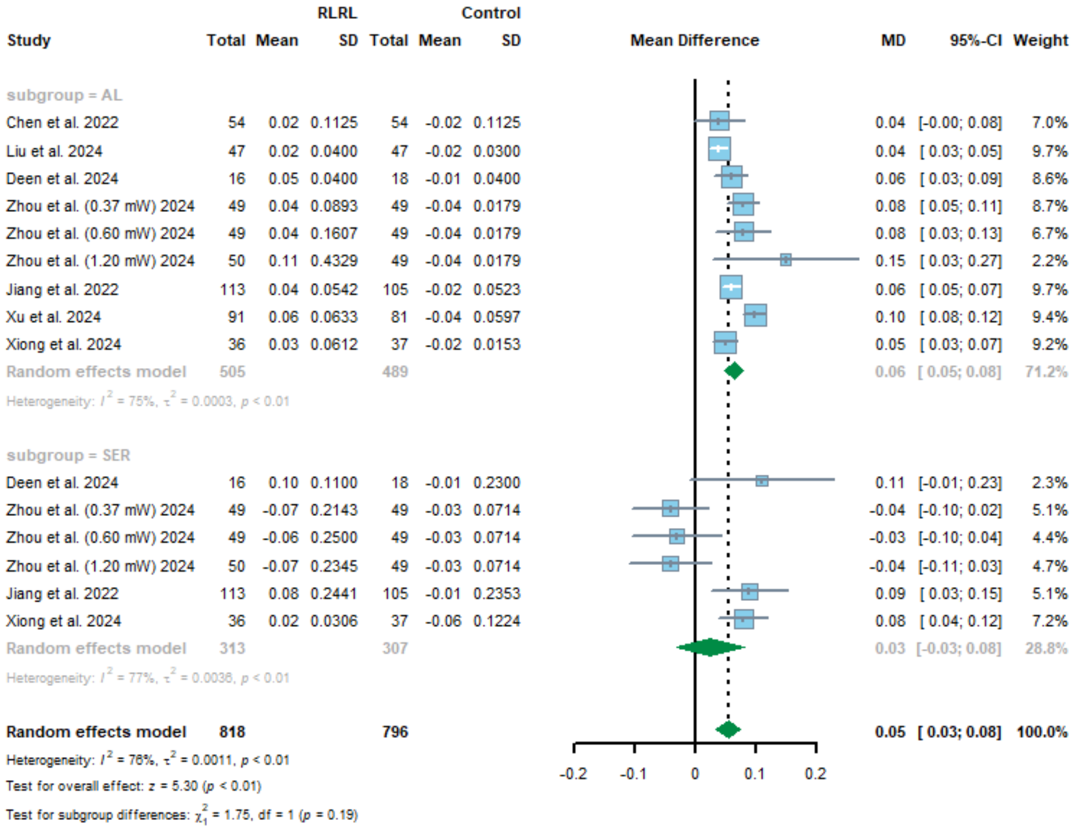

Supplementary figure 5b. Forest plots of AL and SER for 3 month.

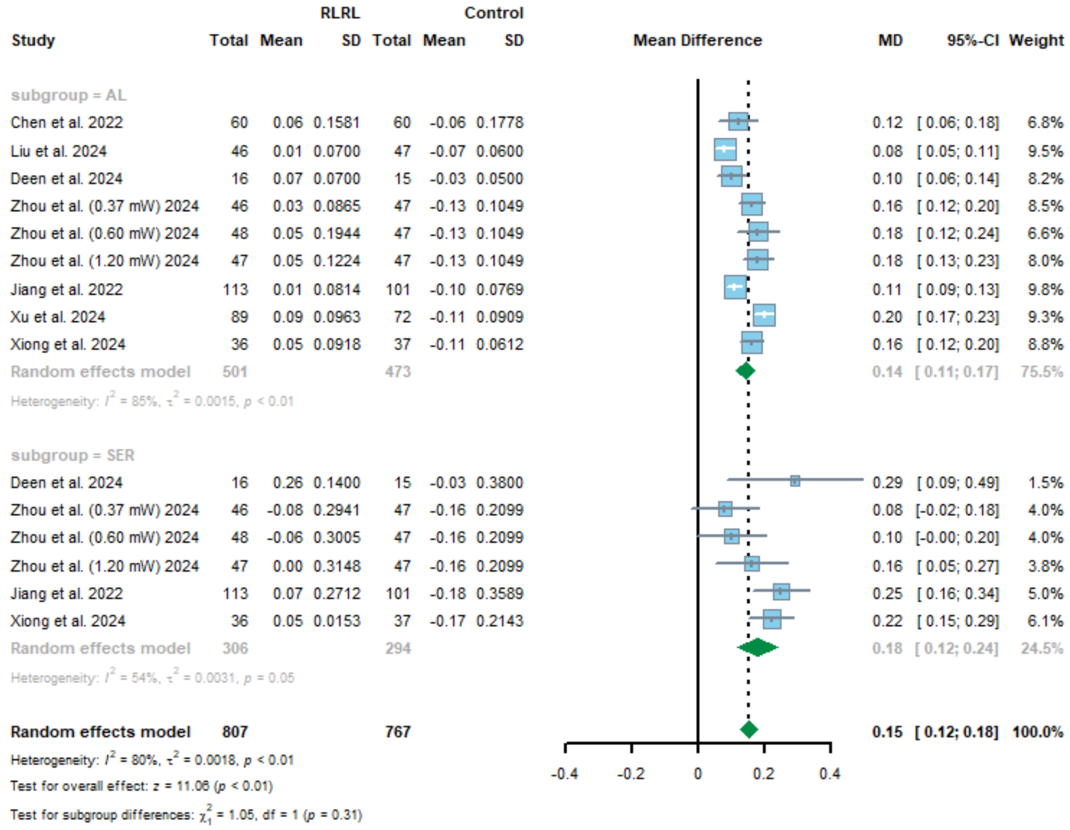

Supplementary figure 5c. Forest plots of AL and SER for 6 month.

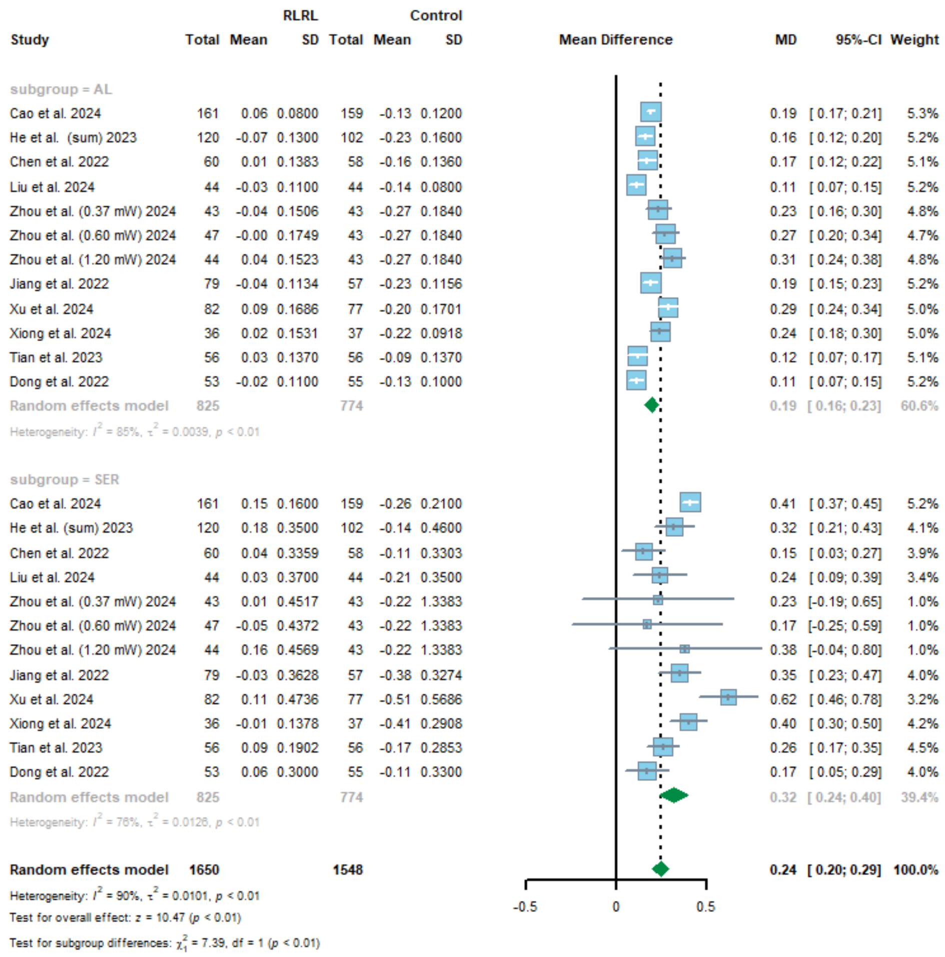

Supplementary figure 5d. Forest plots of AL and SER for 12 month.
