# Supplementary material for: Effects of repeated low-level red light on refractive development during childhood: a systematic review and dose–response meta-analysis up to 12 months
Source: Front Med (Lausanne). 2025 Dec 10;12:1657295. doi: 10.3389/fmed.2025.1657295 (PMC12728021; doi:10.3389/fmed.2025.1657295)
Supplement: Supplementary file 3 [file Data_Sheet_3.zip › Supplementary file 2.html]

```html


RCS Analysis Summary


# Restricted Cubic Spline (RCS) Analysis Results

## AL Analysis

|  |  |
| --- | --- |
| R²: | 0.5392 |
| Adjusted R²: | 0.4997 |
| F-statistic: | 13.65 |
| p-value: | 4.673e-06 |

## SER Analysis

|  |  |
| --- | --- |
| R²: | 0.6884 |
| Adjusted R²: | 0.6550 |
| F-statistic: | 20.62 |
| p-value: | 2.972e-07 |

## Coefficients Comparison

```
