# Supplementary material for: Effects of repeated low-level red light on refractive development during childhood: a systematic review and dose–response meta-analysis up to 12 months
Source: Front Med (Lausanne). 2025 Dec 10;12:1657295. doi: 10.3389/fmed.2025.1657295 (PMC12728021; doi:10.3389/fmed.2025.1657295)
Supplement: Supplementary file 4 [file Table_1.docx]

**Supplementary table 1: Baseline characteristics of included studies.**

| **Author** | **Arm** | **Eyes/Patients** | **Age, year** | **Gender**  **F/M** | **AL, mm** | **SER, Diapoter** |
| --- | --- | --- | --- | --- | --- | --- |
| Cao *et al.* 2024 [1] | RLRL | 168/168 | 9.1 ± 2.0 | 90/78 | 23.9 ± 1.1 | -1.4 ± 1.6 |
|  | No intervention | 168/168 | 9.0 ± 1.9 | 86/82 | 23.8 ± 1.0 | -1.3 ± 1.5 |
| He *et al.* 2023 [2] | RLRL | 139/139 | 8.28 ± 1.10 | 68/71 | 23.36 ± 0.68 | 0.14 ± 0.30 |
|  | No intervention | 139/139 | 8.31 ± 1.07 | 71/68 | 23.30 ± 0.69 | 0.16 ± 0.28 |
| Chen *et al.* 2022 [3] | RLRL | 62/31 | 9.78 ± 1.58 | 17/14 | 24.48 ± 0.79 | -2.60 ± 1.17 |
|  | LDA^1^ | 62/31 | 10.31 ± 1.90 | 14/17 | 24.67 ± 0.98 | -2.59 ± 1.24 |
| Liu *et al.* 2024 [4] | RLRL | 47/47 | 8.98 ± 1.31 | 19/24 | 23.57 ± 0.78 | 0.17 ± 0.35 |
|  | No intervention | 47/47 | 8.95 ± 1.52 | 20/22 | 23.30 ± 0.73 | 0.30 ± 0.35 |
| Deen *et al.* 2024 [5] | RLRL | 43/43 | 11.5 ± 1.55 | 19/24 | 24.2 ± 0.69 | -2.18 ± 0.86 |
|  | No intervention | 16/16 | 11.9 ± 1.39 | 7/9 | 24.6 ± 0.75 | -2.24 ± 1.06 |
| Zhou *et al.* 2024 [6] | RLRL (0.37 mW) | 43/43 | 8.51 ± 1.51 | 16/27 | 24.24 ± 0.81 | -1.79 ± 0.96 |
|  | RLRL (0.60 mW) | 47/47 | 8.77 ± 1.43 | 22/25 | 24.11 ± 0.89 | -2.05 ± 0.88 |
|  | RLRL (1.20 mW) | 44/44 | 8.68 ± 1.39 | 20/24 | 24.38 ± 0.9 | -2.10 ± 1.36 |
|  | No intervention | 43/43 | 8.83 ± 1.53 | 17/26 | 24.44 ± 0.93 | -2.09 ± 0.9 |
| Jiang *et al.* 2022 [7] | RLRL | 119/119 | M10.4 (8.0-13.0)^2^ | 62/57 | 24.54 ± 0.67 | -2.49 ± 0.92 |
|  | No intervention | 145/145 | M10.5 (8.1-13.0)^2^ | 72/73 | 24.62 ± 0.86 | -2.67 ± 1.06 |
| Xu *et al.* 2024 [8] | RLRL | 97 | 10.40 ± 2.4 | 34/63 | 25.93 ± 1.03 | -5.88 ± 1.69 |
|  | No intervention | 95 | 11.20 ± 2.1 | 51/44 | 25.72 ± 0.87 | -5.75 ± 1.17 |
| Liu *et al.* 2024 [9] | RLRL (myopia) | 32 | 9.37±1.69 | 16/16 | 24.71 ± 0.92 | -2.91 ± 1.27  0.36 |
|  | RLRL (premyopia) | 40 | 8.95±0.87 | 22/18 | 23.40 ± 0.63 | 0.36 ± 0.32 |
|  | n-i (myopia) | 36 | 9.55±1.13 | 17/19 | 24.58 ± 0.64 | -2.61 ± 0.98 |
|  | n-i (premyopia) | 36 | 8.94±1.09 | 17/19 | 23.30 ± 0.78 | 0.37 ± 0.30 |
| Xiong *et al.* 2024 [10] | LRL | 36/36 | 8.83±2.06 | 19/17 | 24.38±0.87 | -2.47±1.39 |
|  | No intervention | 37/37 | 9.00±2.00 | 15/22 | 24.47±0.58 | -2.22±0.72 |
| Tian *et al.* 2023 [11] | LLRL | 56/56 | 7.7±1.1 | 33/23 | 23.1±0.8 | 0.25(-0.25, 0.75)^3^ |
|  | No intervention | 56/56 | 7.9±1.8 | 31/25 | 23.1±0.7 | 0.25 (0.00, 0.75)^3^ |
| Dong *et al.* 2022 [12] | RLRL | 56/56 | 10.3 ± 2.07 | 30/26 | 24.7 ± 1.04 | –3.13 ± 1.91 |
|  | Sham device | 56/56 | 9.86 ± 1.41 | 26/30 | 24.6 ± 0.96 | –2.82 ± 1.86 |

^1^ low-dose atropine (LDA); ^2^ Interquartile range (IQR); ^3^ Cumulative adjusted mean (95% CI).
