# Supplementary material for: Effects of repeated low-level red light on refractive development during childhood: a systematic review and dose–response meta-analysis up to 12 months
Source: Front Med (Lausanne). 2025 Dec 10;12:1657295. doi: 10.3389/fmed.2025.1657295 (PMC12728021; doi:10.3389/fmed.2025.1657295)
Supplement: Supplementary file 5 [file Table_2.docx]

**Supplementary table 2: Intervention of included studies.**

| No. | Aurthor | Year | Intervention |
| --- | --- | --- | --- |
| 1 | Cao *et al.* | 2024 | Children in the LLRL group were given a head-worn device with a 650-nm single-wavelength light source incorporated. This device was confirmed to be safe and was certified by the State Administration for Market Regulation of China. Children were expected to use the device 3 minutes twice daily 4 or more hours apart. In both groups, children with myopia could wear single-vision spectacle lenses. No other intervention was provided to the control group. |
| 2 | He *et al.* | 2023 | Children in the intervention group received the RLRL intervention, while those in the control group did not. The RLRL intervention was provided by a desktop device (Eyerising, Suzhou Xuanjia Optoelectronics Technology), which consists of semiconductor laser diodes and delivers low-level red light with a mean (SD) wavelength of 650 (10) nm. The device is certified as a class IIa device by the China National Medical Products Administration. The RLRL intervention was conducted twice per day, 5 days per week, with each session lasting 3 minutes and with an interval of at least 4 hours between 2 sessions. |
| 3 | Chen *et al*. | 2022 | The intervention group received RLRL therapy with a table-mountable commercially available device (Eyerising; Suzhou Xuanjia Optoelectronics Technology, Jiangsu, China). The device emits red light at 650 ± 10 nm from semiconductor laser diodes at an illuminance level of 1600 lux from pupil to fundus. The device was given free of charge to the children, and children and parents were instructed to use the RLRL therapy for 3 minutes twice daily, with at least 4 hours between sessions, for 7 days a week until the last follow-up visit |
| 4 | Liu *et al.* | 2024 | Children in the RLRL therapy group received RLRL treatment twice daily, with each session lasting 3 min and at least 4 h between treatments, while those in the control group did not. The RLRL intervention was provided by a desktop device (Eyerising, Suzhou Xuanjia Optoelectronics Technology, Kunshan city, China). This technology incorporates semiconductor laser diodes that emit low-intensity, single-wavelength 650 (10)-nm red-light laser beam at an illuminance level of approximately 1600 lx through the pupil to the fundus. This device was certified as a Class IIa medical device by the State Administration for Market Regulation of China. The light power of this RLRL device entering a 4-mm pupil (the maximum pupil size under the condition of bright-light exposure over 10 s) is 0.29 mW. |
| 5 | Deen *et al.* | 2024 | The study employed a semi-conductor laser product (Eyerising International Pty Ltd, Melbourne, Australia) emitting low-level red light at 650 ± 10 nm, with a power output of 2.00 ± 0.50 mW and a laser spot diameter at the observation port of 10 mm ± 2 mm. Administered treatment sessions following a specific schedule: 3-minute sessions twice a day, with a minimum 4-hour interval, 5 days a week. Children in the intervention group, alongside their routine activities and wearing single vision spectacles, underwent the therapy regimen under parental supervision. They were reminded to keep their eyes open during the 3-minute sessions and were discouraged from leaving the device. Conversely, the control group received no red-light therapy, continuing routine activities with single vision spectacles for myopia correction. No other myopia interventions were administered to either group for the duration of their participation in the trial. |
| 6 | Zhou *et al.* | 2024 | Participants in this study were assigned randomly to 3 intervention groups and 1 control group at an allocation ratio of 1:1:1:1. Children in the intervention group wore a single-vision spectacle (SVS) throughout the day and were assigned randomly to receive LRL therapy at different powers (sky-n1201; Beijing Ming Ren Shi Kang Science & Technology Co., Ltd.). The LRL device was certified by the China National Medical Products Administration (register no. 20182190267) for treating myopia progression. The light source of the equipment was made with 3 different power settings: power of 0.37 ± 0.02 mW and wavelength of 650 nm, power of 0.60 ± 0.2 mW and wavelength of 650 nm, and power of 1.20 mW and wavelength of 650 nm. Participants took the device home and received treatments twice daily for 3 minutes per session, with at least a 4-hour interval between sessions, under the supervision of their parents. Children in the control group wore an SVS throughout the day and could receive updates if necessary. Best-corrected spectacles were prescribed for all participants throughout the study. |
| 7 | Jiang *et al.* | 2022 | As the standard treatment for optical correction of myopia, all children wore SVSs throughout the study and updated their spectacles if needed. In addition to SVS, children in the intervention group additionally received RLRL therapy. This treatment was provided by a desktop light therapy device (Eyerising [Suzhou Xuanjia Optoelectronics Technology]; Fig S1, available at www.aaojournal.org), which has been on the market and used widely for amblyopia treatment for the past decade in China. This device is certified as a class IIa device by the China National Medical Products Administration (register number, 170808-01039). It consists of semiconductor laser diodes, which deliver low-level red light with a wavelength of 650 ± 10 nm at an illuminance level of approximately 1600 lux through the pupil to the fundus. Children in the RLRL group brought the device home, where they were instructed to complete treatment under supervision of their parents twice daily with an interval of at least 4 hours, with each treatment lasting 3 minutes, during weekdays (5 days per week). This treatment was repeated daily during weekdays until the last follow-up visit at 12 months. |
| 8 | Xu *et al.* | 2024 | According to a routine treatment for myopia, all participants in both groups wore SVS lenses throughout the study. Besides wearing SVS lenses, the intervention group underwent RLRL treatment using a portable desktop device (Eyerising International). The device incorporates a semiconductor laser diode capable of emitting low-level red light with a wavelength of 650 nm and a luminance of 1600 lx through the pupil to the retina. At the retina, the power measures 0.29 mW with a pupil diameter of 4 mm. After the baseline evaluation, participants in the intervention group started the RLRL treatment twice a day, 7 days a week, until the 12-month follow-up, with each session lasting 3 minutes. |
| 9 | Liu *et al.* | 2024 | A low-level red light therapy device (Eyerising; Suzhou Xuanjia Optoelectronics Technology, Suzhou, China) was used in this study. It consists of semiconductor laser diodes, which deliver low-level red light with a wavelength of 650±10nm at an illuminance level of approximately 1600lux through the pupil to the fundus. Subjects in the RLRL group took the device home, where they were instructed to complete treatment twice daily with an interval of at least 4hours, with each treatment lasting 3min, for 7days per week. Subjects were asked to adjust the best position and wear refractive correction spectacles before use to ensure the light entered the eyes properly. |
| 11 | Xiong *et al.* | 2024 | Participants wore single vision spectacles (SVS) and received low-level red light treatment by a desktop light therapy device (Yishiliang [Hunan Yuanliang health technology Co., LTD. Hunan, China]). It has a 650 nm wavelength with a transmitted power of 0.9 mW, an extended source subtending 3.6°, input power of 0.178 mW for a pupil size of 4 mm, and illuminance level set at 700 lx. After powering on the device, it will automatically emit low-level red light for a duration of 3 min. Subsequent light emission can only be initiated after a lapse of 4 h. Participants will be instructed to take the device home and utilize it under guardian supervision, illuminating both eyes twice daily for a period of 3 min each time. No other measures for myopia control were combined during the intervention. The control group (SVS) exclusively wore fully corrected single vision spectacle without implementing any additional measures for myopia control during the intervention. |
| 12 | Tian *et al.* | 2023 | Children in the treatment group were given a head-worn device called the light feeding apparatus, it incorporates a single wavelength light source (650 nm). Children were expected to use the device to irradiate the retina for six minutes every day, divided into two times of three minutes, with an interval of ≥ 4 h. Children in both groups were allowed to wear a single-vision spectacle lenses (SVS) if they were myopic. No other intervention was given to the control. |
| 13 | Dong *et al.* | 2022 | In addition to SVS, children in the intervention group received RLRL therapy, and those in the sham device control group received sham light therapy, both at a treatment schedule of 3 minutes per session, twice daily, with an interval between sessions of at least 4 hours. The RLRL group received a desktop red-light therapy device (Eyerising; Suzhou Xuanjia Optoelectronics Technology) that has been widely used for amblyopia treatment in China over the past few decades. The sham device control group received the same device but with only 10% of the original device’s power. The light power entering a 4-mm pupil (the maximum pupil size under the condition of bright light exposure over 10 seconds) was 0.29 mW for the RLRL device and 0.03 mW for the sham device. |
